# Prenatal LPS leads to increases in RAS expression within the PVN and overactivation of sympathetic outflow in offspring rats

**DOI:** 10.1038/s41440-024-01754-z

**Published:** 2024-07-05

**Authors:** Xueqin Hao, Xueting Long, Lingling Fan, Jijia Gou, Yuchao Liu, Yifan Fu, Huijuan Zhao, Xiaojuan Xie, Dongmei Wang, Gaofeng Liang, Yujia Ye, Jing Wang, Sanqiang Li, Chunyu Zeng

**Affiliations:** 1 Department of human Anatomy and Histoembryology, College of Basic Medicine and Forensic Medicine, Henan University of Science and Technology, Luoyang, Henan China; 2 Department of Anesthesiology, the First affiliated Hospital of Henan University of Science and Technology, Luoyang, Henan China; 3 Department of Physiology, College of Basic Medicine and Forensic Medicine, Henan University of Science and Technology, Luoyang, Henan China; 4 Department of Microbiology, College of Basic Medicine and Forensic Medicine, Henan University of Science and Technology, Luoyang, Henan China; 5 Department of Pathology, College of Basic Medicine and Forensic Medicine, Henan University of Science and Technology, Luoyang, Henan China; 6 Department of Cardiology, The First Affiliated Hospital of Kunming Medical University, Kunming, China; 7 Department of Biochemistry, College of Basic Medicine and Forensic Medicine, Henan University of Science and Technology, Luoyang, Henan China; 8 Department of Cardiology, Daping Hospital, Third Military Medical University, Chongqing, China

**Keywords:** Prenatal lipopolysaccharide, Hypertension, Sympathetic nervous system, Brain RAS, Melatonin

## Abstract

The renin-angiotensin system (RAS) and the sympathetic nervous system (SNS) are two major blood pressure-regulating systems. The link between the renal and cerebral RAS axes was provided by reflex activation of renal afferents and efferent sympathetic nerves. There is a self-sustaining enhancement of the brain and the intrarenal RAS. In this study, prenatal exposure to lipopolysaccharide (LPS) led to increased RAS activity in the paraventricular nucleus (PVN) and overactivation of sympathetic outflow, accompanied by increased production of reactive oxygen species (ROS) and disturbances between inhibitory and excitatory neurons in PVN. The AT1 receptor blocker losartan and α2 adrenergic receptor agonist clonidine in the PVN significantly decreased renal sympathetic nerve activity (RSNA) and synchronously reduced systolic blood pressure. Prenatal LPS stimulation caused H3 acetylation at H3K9 and H3K14 in the PVN, which suggested that epigenetic changes are involved in transmitting the prenatal adverse stimulative information to the next generation. Additionally, melatonin treatment during pregnancy reduced RAS activity and ROS levels in the PVN; balanced the activity of inhibitory and excitatory neurons in the PVN; increased urine sodium secretion; reduced RSNA and blood pressure. In conclusion, prenatal LPS leads to increased RAS expression within the PVN and overactivation of the sympathetic outflow, thereby contributing to hypertension in offspring rats. Melatonin is expected to be a promising agent for preventing prenatal LPS exposure-induced hypertension.

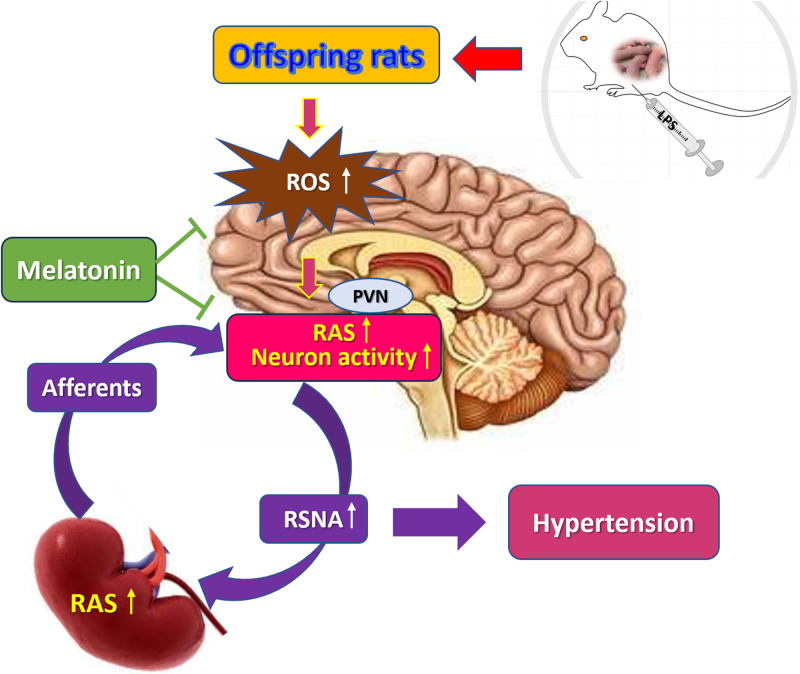

## Introduction

Hypertension is the leading cause of the mortality associated with cardio-cerebrovascular diseases [1]. According to epidemiological and experimental studies, adult hypertension may originate from embryonic and neonatal stages [2]. Adverse conditions in the fetal environment, such as insufficient protein intake or consumption of a high-fat and high-cholesterol diet by the mother during pregnancy [3, 4], and maternal glucocorticoid administration [5], can cause hypertension in offspring. In our previous study, lipopolysaccharide (LPS) administration to pregnant mothers caused hypertension in offspring rats [6]; however, the underlying mechanism remains unclear.

Overactivation of the sympathetic nervous system (SNS) is involved in hypertension pathogenesis [7]. Presympathetic neurons in the paraventricular nucleus of the hypothalamus (PVN) generate and control the sympathetic vasomotor tone [7, 8]. These PVN presympathetic neurons receive both excitatory and inhibitory inputs [8], with the balance of these inputs determining neuron excitability. The PVN presympathetic neurons project to sympathetic premotor neurons in the rostral ventrolateral medulla (RVLM) and sympathetic preganglionic neurons in the mediolateral spinal cord, thereby being a crucial source of excitatory drive for the sympathetic outflow [8]. Mothers with diabetes mellitus are more likely to produce hypertensive offspring with augmented renal sympathetic nerve activity (RSNA) [9]. Moreover, maternal obesity resulted in sympathetic overactivity and hypertension in offspring [10].

The renin-angiotensin system (RAS) is another major system in regulating blood pressure (BP). The RAS and SNS reinforce each other in promoting the onset and development of hypertension [11]. Our previous study shows that maternal LPS administration during pregnancy increased intrarenal RAS activity in offspring rats, independent of the circulating RAS. However, the mechanism through which intrarenal RAS activity increases BP levels remains unclear. Studies have reported that adverse stimulation during pregnancy leads to higher RAS activity in the brain. For example, a high fructose diet consumed by pregnant mothers induces hypertension, and higher expression of the AT1 receptor (AT1R) protein in the RVLM of offspring [12]. Prenatal cold exposure increased AT1R expression in the PVN and sympathetic nerve hyperactivity in offspring rats [13]. Therefore, we suggest that brain RAS overactivity enhances sympathetic activity. The interaction between the brain RAS and SNS caused hypertension in offspring rats.

Oxidative stress is involved in maintaining high BP. In a neurogenic hypertensive model, superoxide dismutase overexpression in the RVLM leads to reduced BP and improved RSNA [7]. Therefore, antioxidant substances that act on the brain may be beneficial for hypertension treatment. Melatonin, a pineal gland-secreted neurohormone [14], is a powerful free radical scavenger and antioxidant [15]. It is specifically efficient in inhibiting brain oxidative damage because of its solubility in lipophilic and aqueous media and its ability to penetrate the blood–brain barrier [15]. In Nishi’s renovascular hypertension model, melatonin significantly reduced ROS in the RVLM, as well as the RSNA and BP. Additionally, it restored the cardiac baroreflex and RSNA reflex to normal in two kidneys, one clip (2K-1C) hypertensive rats [7]. Melatonin is considered an agent with therapeutic potential against hypertension, which it exerts especially through its sympathetic action.

In the 2K-1C hypertensive model, angiotensin II- and oxidative stress-induced increase in the activities of PVN and RVLM neurons is a major mechanism associated with the maintenance of sympathoexcitation of the cardiovascular system [16]. This study verified our hypothesis that prenatal LPS exposure results in RAS activation in the PVN and overactivation of the sympathetic outflow, the RAS and SNS reinforce each other, and the crosstalk between the kidney and brain, which causes hypertension in offspring rats. We examined RAS activity in PVN, synchronously recorded electrical activity of PVN neurons and RSNA, tested ROS levels in the PVN and mesenteric arteries. We also assessed the effects of melatonin on prenatal LPS-induced RAS activity, electrical activity of PVN neurons, RSNA, ROS levels, and hypertension induced by prenatal LPS in offspring rats.

## Materials and methods

### Animals

Forty-five Sprague Dawley rats (weight: 350–380 g) were purchased from Sikebas Biotechnology Co., Ltd. (license number: SCXK (Lu) 20190003). All animals had free access to standard laboratory rat chow and tap water. Ten male and 20 female rats were mated at the 1:2 ratio at room temperature (24 °C ± 1 °C) under a 12-h light–dark cycle. Vaginas of the female rats were examined at 7:00 and 19:00 on the following day. A positive vaginal smear for sperm or having a vaginal plug was defined as day 0 of pregnancy. This study was conducted according to the principles outlined in the National Institutes of Health Guide for the Care and Use of Laboratory Animals (http://grants1.nih.gov/grants/olaw/). The study protocol was approved by the Ethical Committee for Animal Experimentation of Henan University of Science and Technology.

### Experimental design and rat treatments

All pregnant rats were randomly divided into four groups. The LPS and LPS + MT groups received intraperitoneal injections of 0.79 mg/kg LPS on days 8th, 10th, and 12th of pregnancy. The control group received intraperitoneal injections with the same volume of sterile saline. The MT and LPS + MT groups were intragastrically administered melatonin (30 mg/kg) daily from day 0 to day 16 of gestation. After the offspring rats were born, they were breastfed for 1 month and then fed with the standard diet. Eight offspring rats in each group were randomly used for the experiment.

### Measurement of non-invasive systolic BP

Systolic BP (SBP) was measured at 15:00 to 18:00 once every 3 weeks from 8 to 26 weeks of age in offspring rats by using the standard tail-cuff method (ML125; Powerlab, AD Instruments, Castle Hill, NSW, Australia). Before SBP measurement, the rats were placed inside a warming chamber (~34 °C) for 15 min and then in a plastic restraint. A cuff with a pneumatic pulse sensor was attached to the tail. The mean SBP was calculated using five consecutive SBP recordings in each rat. The rats had been trained at least three times before the formal measurement. Both male and female offspring rats were included in the present study.

### Urine collection

The rats were placed in metabolic cages for habituation for 1 day before urine collection was started. The feed was ground into a powder with a machine and then mixed with water to form a paste. During urine collection, the rats in each group were allowed free consumption of the food in the form of a paste and water at an ambient temperature of approximately 24 °C. One rat was placed in the metabolic cage for 24 h for urine collection. Then, the urine was centrifuged at 2500 × *g* for 10 min at 4 °C, and the supernatant was used for measuring 24-h urine sodium and potassium. The urine sodium and potassium levels were measured in the clinical lab in the First Affiliated Hospital of Henan University of Science and Technology.

### Invasive BP measurement

Rats were anesthetized with sodium pentobarbital (45 mg/Kg, i.p.) and placed on a suitable rodent surgical table. A 1.5- to 2-cm small incision was made in the skin of the inguinal region. The femoral artery was identified and separated from the adjacent connective tissue and femoral vein. The distal part of the blood vessel was ligated, while its proximal part was clamped with an artery clamp for cannulation. The cannula was inserted into the artery and tied with a thread without obstructing the blood flow in the femoral cannula. The other end of the cannula was connected to a three-way stopcock that was connected to the pressure transducer and a syringe filled with heparinized saline (5 IU/mL).

### Recording of RSNA

The left kidney, renal artery, and renal nerve were exposed along the retroperitoneal path after creating a longitudinal lumbar incision. The renal sympathetic nerve was carefully freed near the renal artery and renal vein close to the abdominal aorta and immersed in paraffin oil. Silver wire electrodes were placed to guide RSNA. After amplification using the AC/DC differential amplifier (Model 3000, A-M System Inc, USA) (low cut-off frequency at 60 Hz, high cut-off frequency at 2 kHz), RSNA was recorded using the PowerLab data analysis and processing system (Model 8SP, AD Instruments, Australia) and integrated. The raw RSNA values and RSNA integral values were simultaneously recorded. At the end of the experiment, the central end of the renal nerve was cut to eliminate renal sympathetic outflow activity. The noise level was recorded, and the actual RSNA integral value was calculated by subtracting the noise integral value from the RSNA integral value. Arterial BP and RSNA were recorded simultaneously by using the PowerLab data analysis and processing system. BP was recorded in channel 1, the raw RSNA was recorded in channel 2, and the raw RSNA was integrated forward in channel 3. Data was analyzed using the PowerLab system.

### Extracellular single-unit recording in vivo

Each rat was anesthetized and placed on a heated board to maintain the body temperature at 37 °C. The adequate depth of anesthesia was assessed based on the absence of corneal reflexes and paw withdrawal response to a noxious pinch, and this depth was maintained during the experiment by administering supplemental doses of anesthesia. The right femoral artery was cannulated for recording arterial BP. The electrical activity of PVN neurons was determined from the extracellular single-unit recordings gathered in vivo by using the PowerLab data analysis and processing system (Model 8SP, AD Instruments, Australia). The stereotaxic coordinates for the PVN (AP 1.6–1.8 mm, L 0.4–0.6 mm, D 7.2–8.2 mm) were determined according to Paxinos and Watson’s atlas. Extracellular single-unit recordings were gathered using a glass microelectrode (tip impedance: 8–16 MΩ) filled with 0.5 M sodium acetate containing 2% pontamine sky blue. The microelectrode was deepened into the PVN by using a micropropulsion controller. The spontaneous neuron activity was amplified using the AC/DC differential amplifier with a low-frequency cut-off at 30 Hz and a high-frequency cut-off at 3 kHz.

### PVN injection

The head of an anesthetized rat was mounted on a stereotaxic apparatus (Model 900, David Kopf Instruments, Tujunga, CA, USA). The coordinates of bregma and lambda were examined, and the head was adjusted to be horizontal. After a midline scalp incision was made to expose the periosteum, a 4-mm-diameter hole was drilled in the skull by using a high-speed drill. Using a microliter syringe (diameter, 50 μm), intracerebral injections were administered into the PVN (1.8 mm posterior, 0.4 mm lateral from the midline, and 7.9 mm ventral). Losartan (10 μg/kg) was injected to block the AT1 receptor in the PVN. Clonidine (20 μg/kg), an a2 adrenergic receptor agonist, was injected to diminish the efferent sympathetic tone. The control rats were injected with the same volume of saline in the PVN.

### Immunofluorescence

The rats were perfused with 4% paraformaldehyde phosphate-buffered saline through the left cardiac ventricle. The brain tissues were fixed in 4% paraformaldehyde for 48 h and dehydrated in 30% sucrose solution at 4 °C for 48 h to sediment. Then, the tissues were embedded in the Tissue-Tek OCT compound (Miles, Elkhart, IN, USA) and frozen for 30 min in a −80 °C freezer. Twenty-micron frozen sections were cut and mounted on gelatin-coated slides. After the brain slices were blocked with 5% goat serum in 0.3% Triton X-100 for 60 min at room temperature, they were incubated at 4 °C overnight with the following primary antibodies: rabbit anti-AT1 receptor (1:100, Proteintech, Wuhan, China), rabbit anti-GRIN2B antibody (excitatory glutamatergic neuron marker, 1:100, Proteintech, Wuhan, China) and rabbit anti-GAD65 antibody (inhibitory GABAergic neurons marker, 1:100, Proteintech, Wuhan, China), rabbit anti-H3K9ac (1:400, #9649, Cell Signaling Technology, Danvers, MA, USA), anti-H3K27ac (1:100, #8173, Cell Signaling Technology, Danvers, MA, USA), and anti-H3K14ac (1:10000, #7627, Cell Signaling Technology, Danvers, MA, USA). The slices were then incubated with the appropriate fluorescence-conjugated secondary antibody (CoraLite488-conjugated Goat Anti-Rabbit lgG(H + L), 1:1000, Proteintech, Wuhan, China) and (Rhodamine(TRITC)-conjugated Goat Anti-Rabbit lgG(H + L), 1:100, Proteintech, Wuhan, China) at 37 °C for 1 h. Images were captured using an inverted fluorescence microscope (TE-2000U, Nikon, Tokyo, Japan).

### Dihydroethidium staining

The rats were anesthetized with sodium pentobarbital (45 mg/kg, i.p.) and injected with dihydroethidium (DHE) (5 mg/kg, Beyotime Biotech, Shanghai, China) through the femoral vein. The rat brains and mesentery arteries were rapidly removed and frozen at −80 °C. To detect the ROS level in the brain tissues, brain slices (thick: 20 µm) were prepared using a cryostat (Leica CM1850, Leica Biosystems, Wetzlar, Germany). Images were captured using an inverted fluorescence microscope (TE-2000U, Nikon, Japan), and the DHE-positive cells were quantified using Image-PRO Plus 5.0 (Media Cybernetics, Silver Spring, MD, USA).

### Statistical analysis

Data were presented as mean ± SE. Between-group differences were determined by applying a two-way ANOVA, followed by the Newman-Keuls test for the post hoc analysis of significance (StatView II, Berkeley, CA). *P* < 0.05 was considered statistically significant.

## Results

### Prenatal LPS resulted in hypertension in offspring rats

**Fig. 1:**
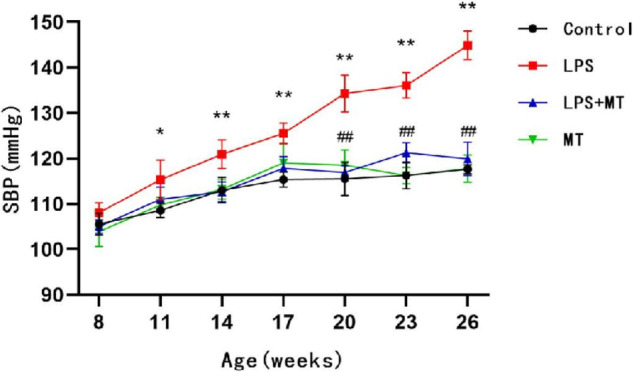
Prenatal LPS resulted in hypertension in offspring rats. SBP were recorded by tail-cuff method in conscious offspring rats at 7–26 weeks of age (^*^P < 0.05, ^**^P < 0.01 compared with Control group, ^##^*P* < 0.01 compared with L*P*S group, *n* = 10 in each group)

The SBP of offspring rats in the LPS group increased gradually from 11 weeks of age and reached 144.9 mmHg at 26 weeks of age. This SBP value was significantly higher than those in the control and LPS + MT groups. No significant difference was observed between the control and MT groups (Fig. 1). This suggests that prenatal LPS exposure causes hypertension in offspring rats, and melatonin treatment during pregnancy effectively protected the offspring rats from prenatal LPS stimulation-induced hypertension.

### Prenatal LPS resulted in decreased sodium excretion in offspring rats

Urine was collected from 24 households of 26-week-old offspring rats to examine urinary sodium and potassium levels. At 26 weeks of age, the 24-h urinary sodium content of the offspring of LPS-treated rats was lower than that of the control rats. However, the 24-h urinary potassium content of the offspring of the LPS-treated rats was higher than that of the control rats. After being treated with melatonin, the LPS + MT group exhibited a significant increase in urinary sodium levels, with a decrease in urinary potassium content (Table 1).

**Table 1:** Effects of prenatal LPS stimulation on urinary sodium and potassium in adult offspring rat

	24-h urine Na	24-h urine K	24-h urine Na/K
Control	0.65 ± 0.01	2.86 ± 0.06	0.22 ± 0.01
LPS	0.34 ± 0.01^**^	3.86 ± 0.09^*^	0.08 ± 0.01^**^
LPS + MT	0.51 ± 0.05^#^	2.80 ± 0.27^#^	0.19 ± 0.02^#^
MT	0.52 ± 0.01	2.64 ± 0.07	0.19 ± 0.01

24-h urine was collected in offspring rats at 7–26 weeks of age

^*^P < 0.05, compared with Control group; ^#^*P* < 0.05, compared with LPS group

### Increased RSNA and electrical activity of PVN neurons in prenatal LPS-exposed offspring rats

**Fig. 2:**
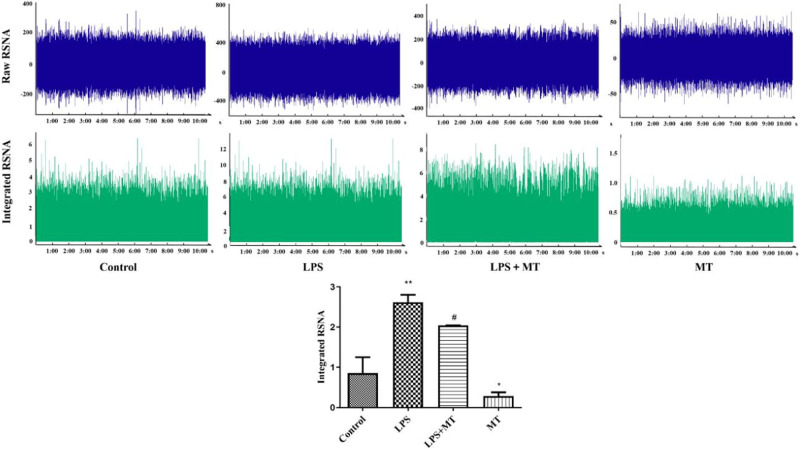
Prenatal LPS resulted in increased renal sympathetic nerve activity in adult offspring rats. Renal sympathetic nerve activity was recorded, and integral data was processed by Powerlab system (^*^P < 0.05, ^**^P < 0.01 compared with Control group, ^#^*P* < 0.05 compared with L*P*S group, *n* = 8)

**Fig. 3:**
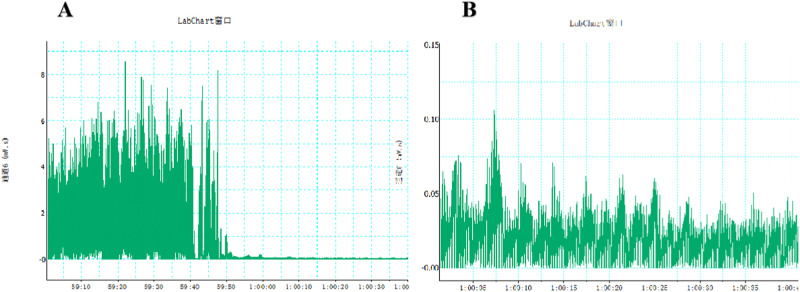
Cut off the central end of renal nerve to eliminate renal sympathetic outflow activity and record the noise level. **A** The sympathetic nerve activity was significantly reduced when the renal sympathetic nerve was cut off a period of time after recording of renal sympathetic nerve activity; **B** The recorded ambient noise level after cutting off the renal sympathetic nerve

We synchronously examined the RSNA and electrical activity of PVN neurons. Compared with the control group, the LPS group had higher RSNA. Melatonin significantly reduced LPS-induced renal sympathetic nerve hyperactivity (Fig. 2). The sympathetic nerve activity was significantly reduced when the renal sympathetic nerve was cut off a period of time after recording of renal sympathetic nerve activity(Fig. 3).

**Fig. 4:**
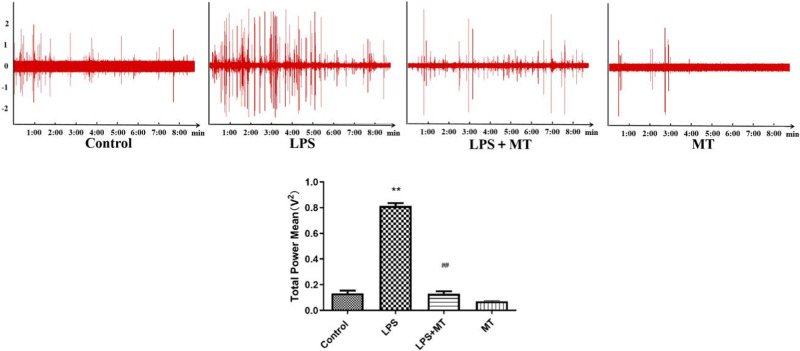
Discharge patterns and electrical activity of neurons in the PVN of offspring rats. Electrical activity of PVN neurons was expressed as total power mean value recorded from extracellular single-unit in vivo using glass microelectrodes. (^**^P < 0.01 compared with Control group, ^##^*P* < 0.01compared with LPS group, *n* = 8)

The total power level of PVN neurons in the offspring of the LPS-treated rats was higher than that in the offspring of the control rats. Melatonin treatment significantly reduced the total power mean value in response to LPS exposure (Fig. 4).

**Fig. 5:**
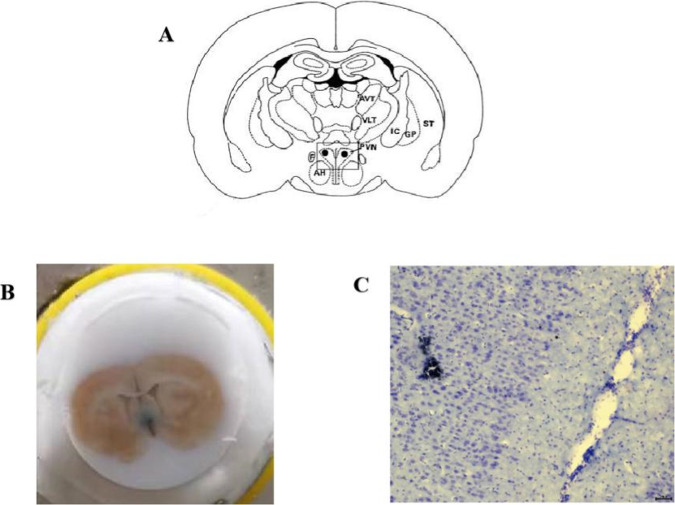
Determination of PVN discharge location. **A** Location of PVN in Paxinos and Watson’s atlas; **B** Electrophoretic Pontamine Sky Blue; **C** Nissl staining showing recording sites labeled with Pontamine Sky Blue in PVN

After attaining the electrophysiological recordings, the last recording site was labeled through glass microelectrode electrophoresis with Pontamine Sky Blue (Fig. 5).

### Overactivation of sympathetic outflow is involved in prenatal LPS exposure-induced hypertension in offspring rats

**Fig. 6:**
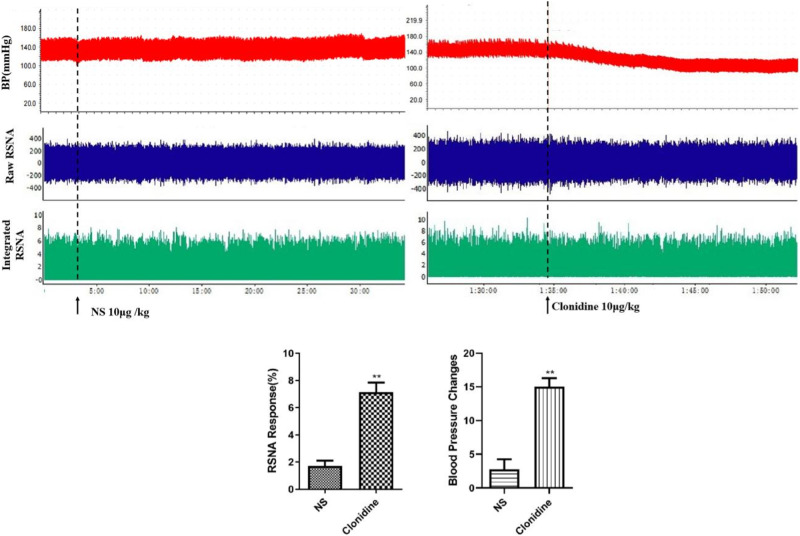
Microinjection of clonidine into hypothalamic PVN reduced blood pressure and renal sympathetic nerve activity in prenatal LPS-exposed offspring rats. Rats were anesthetized with pentobarbital, renal sympathetic discharge was recorded by Powerlab system, and femoral arterial blood pressure were measured from the femoral artery (^**^P < 0.01 compared with NS group, *n* = 8)

Clonidine is a central antihypertensive drug that activates α2 receptors. BP and RSNA in the prenatal LPS-exposed offspring rats after clonidine (10 μg/kg) microinjection into the PVN were significantly lower than those after the rats were injected with the same dose of saline (Fig. 6). Thus, sympathetic nerve hyperactivity was the key cause of prenatal LPS stimulation-induced hypertension in offspring rats.

### Higher central RAS activity and imbalance between inhibitory and excitatory neurons in PVN led to hypertension in offspring rats

**Fig. 7:**
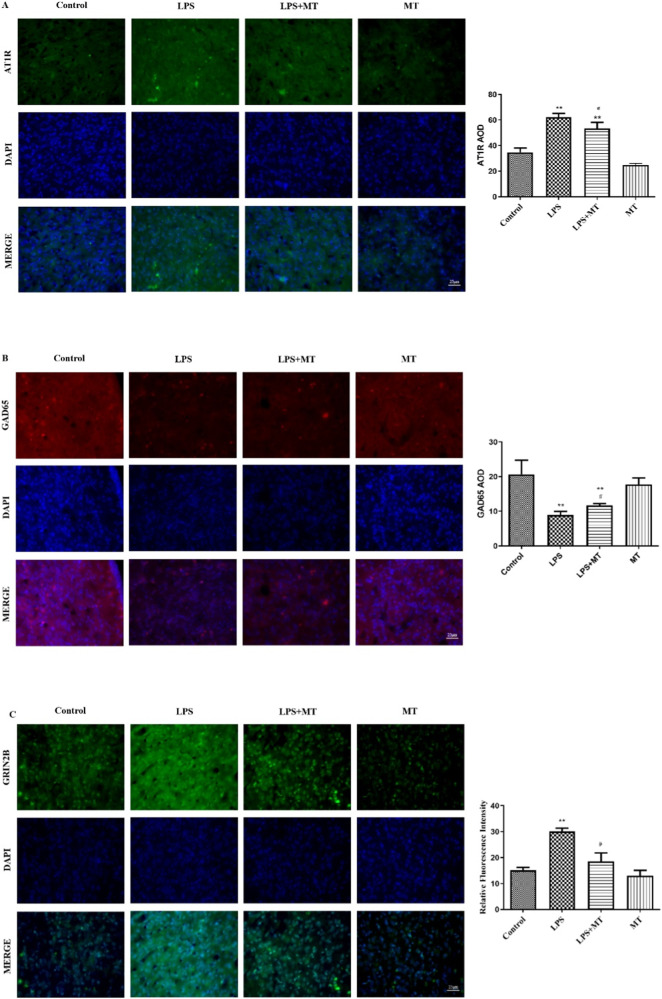
Effects of prenatal LPS on the protein expressions of AT1R, GAD65 and GRIN2B in PVN in adult offspring rats. AT1R (**A**), GAD65 (**B**) and GRIN2B (**C**) protein expression was detected by Immunofluorescence. The staining in each group was repeated at least four times, and ten fields of vision in each slide were chosen for AOD assessment (^*^P < 0.05, ^**^P < 0.01 compared with Control group, ^#^*P* < 0.05, ^##^*P* < 0.01 compared with LPS group^,^ *n* = 8)

To investigate the effect of prenatal LPS on central RAS and RSNA, AT1R protein expression was first examined in the PVN. AT1R protein expression was higher in the prenatal LPS-exposed offspring rats than in the control offspring rats, which suggested an augmented central RAS activity in the offspring of the LPS group (Fig. 7A). We then tested the numbers of inhibitory and excitatory neurons in the PVN. The prenatal LPS-exposed offspring rats had decreased numbers of inhibitory GABAergic neurons (Fig. 7B), whereas increased numbers of glutamatergic excitatory neurons in the PVN (Fig. 7C). Melatonin treatment alleviated these changes in the offspring rats.

### Enhanced central RAS activity is key for augmented RSNA induced by prenatal LPS stimulation

**Fig. 8:**
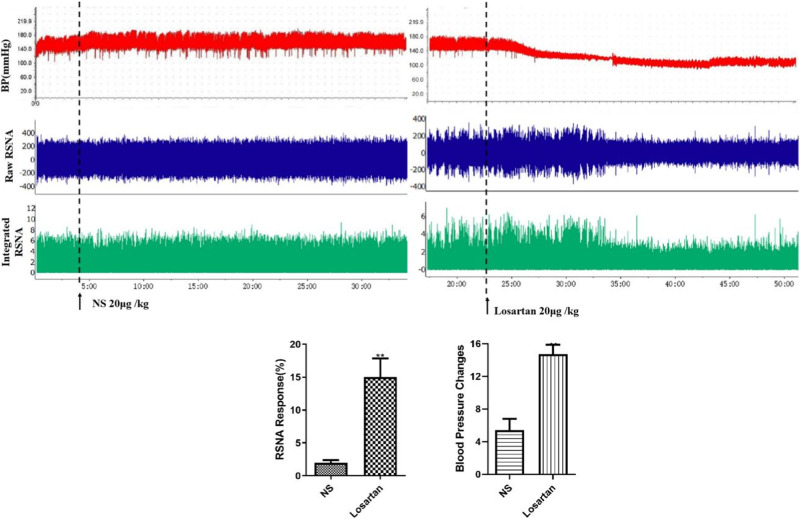
Microinjection of Losartan into hypothalamic PVN reduced blood pressure and renal sympathetic nerve activity (^**^P < 0.01 compared with NS group, *n* = 8)

Losartan, an AT1 receptor blocker (20 μg/kg), microinjected into the PVN of the hypothalamus significantly decreased BP and RSNA in the prenatal LPS-exposed offspring rats. These results suggested that increased central RAS activity is the cause of prenatal LPS exposure-induced augmentation of the RSNA (Fig. 8).

### Melatonin reduced ROS in the PVN and mesenteric arteries in prenatal LPS-exposed offspring rats

**Fig. 9:**
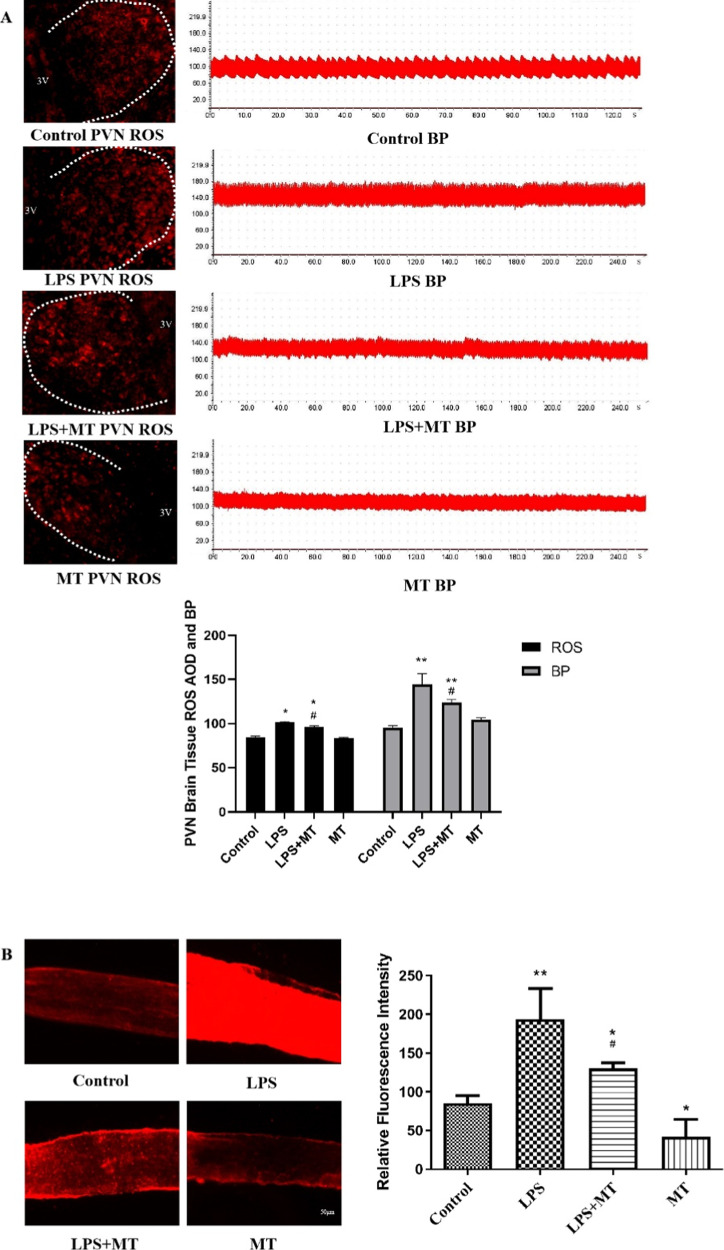
Melatonin decreased prenatal LPS-induced ROS production both in PVN (**A**) and mesenteric arteries (**B**) in adult offspring rats. The ROS level in PVN was positively correlated with blood pressure (**A**). The ROS was detected by DHE staining (^*^P < 0.05, ^**^P < 0.01 compared with Control group; ^#^*P* < 0.05 compared with L*P*S group, *n* = 8)

ROS levels in the PVN and mesenteric arteries were examined to investigate the effect of melatonin on prenatal LPS exposure-induced ROS levels and hypertension. The offspring of the LPS-treated rats had significantly higher ROS levels than the control offspring rats both in the PVN (Fig. 9A) and mesenteric arteries (Fig. 9B). The ROS levels were also positively correlated with BP(Fig. 9A). Melatonin treatment significantly eliminated the ROS levels in the PVN and mesenteric arteries.

### Upregulation of H3K9ac and H3K14ac contributed to the inheritance of epigenetic characteristics of LPS stimulation from the mother to offspring

**Fig. 10:**
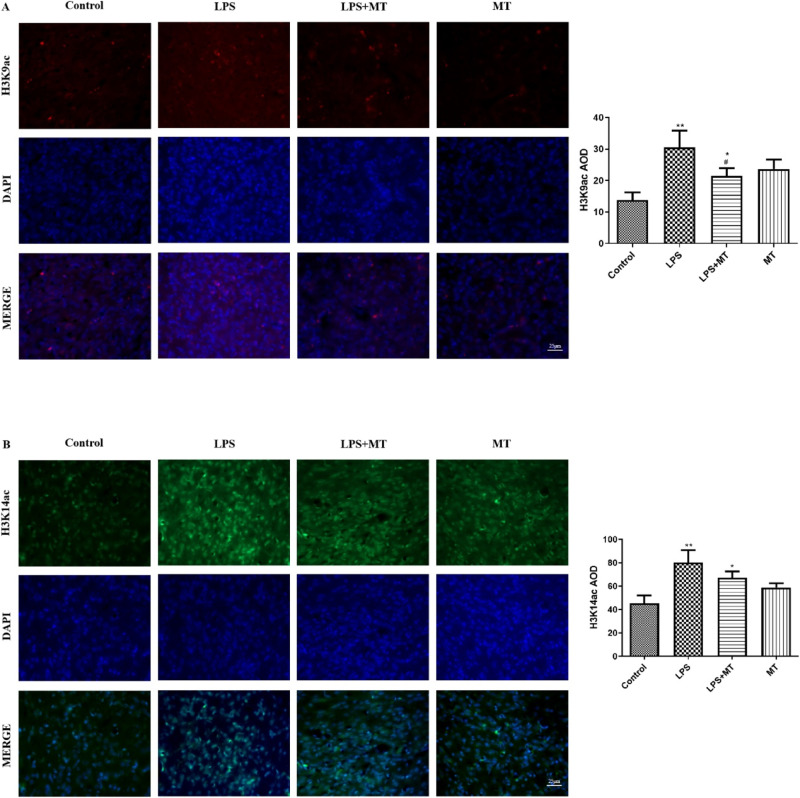
Prenatal LPS exposure increased H3K9ac and K3K14ac protein expression in offspring rats. **A** Results showed the expression of H3K9ac(red) in PVN in offspring rats. DAPI (blue) staining nuclei. **B** Results showed the expression of H3K14ac (green) in offspring rats. DAPI (blue) stained nuclei. (^*^P < 0.05, ^**^P < 0.01 compared with Control group, ^#^*P* < 0.05 compared with LPS group, *n* = 8)

To investigate how the adverse information of LPS stimulation was transferred from the parents to the next generation, we examined H3 acetylation at H3K9, H3K14, and H3K27. H3K9ac(red) and H3K14ac protein expression significantly increased in the prenatal LPS-exposed offspring rats compared with the control offspring rats. Melatonin treatment reduced H3K9ac expression (Fig. 10A) in the prenatal LPS-exposed offspring rats, but no change was noted in H3K14 expression (Fig. 10B).

## Discussion

In the present study, prenatal LPS exposure significantly increased oxidative stress, RAS activity in the PVN region, central sympathetic outflow, RSNA, and hypertension in offspring rats. Prenatal administration of melatonin ameliorated BP by suppressing brain RAS and sympathetic outflow in the offspring rats.

Ang-II is the main effector peptide of RAS that acts predominantly at AT1R to impact BP [17]. The brain has its own intrinsic RAS with the major components [17, 18]. The PVN is a crucial integrative center controlling BP and sympathetic outflow. The PVN neurons are divided into magnocellular and parvocellular subnuclei [17]. The activation of magnocellular neurons primarily leads to the release of neuropeptides vasopressin and oxytocin from the posterior pituitary into the systemic circulation. On the other hand, some magnocellular neurons project into the spinal cord, thereby regulating SNS activity and influencing BP. Parvocellular neurons in the PVN are also classified into two types, namely pre-autonomic and neurosecretory, which control the sympathetic tone and neuroendocrine secretion, respectively [17]. AT1Rs are located in the postsynaptic element of the preautonomic glutamate neurons in the PVN and directly activate these neurons to sustain increases in the SNS activity [17].

Our previous study unveiled that prenatal LPS administration activates intrarenal RAS and renal damage in the offspring rats [19]. In Wilcox’s study, renal and cerebral RAS were linked and self-reinforced mutually through a reflex activation of renal afferents and efferent sympathetic nerves [20]. In the present study, prenatal LPS exposure increased the central sympathetic outflow and the peripheral RSNA. Administration of the selective α2 adrenergic receptor agonist clonidine or the AT1R blocker losartan in the PVN synchronously reduced BP and decreased RANA. Thus, brain RAS activation promotes the overactivation of central sympathetic outflow and the peripheral RSNA.

GABAergic inhibitory and glutamatergic excitatory inputs within the PVN serve a crucial role in controlling sympathetic outflow [8, 21]. GABA is primarily regulated by the isoform of glutamic acid decarboxylase (GAD), that is, GAD65. This enzyme increases GABA levels which helps blunt the GABAergic deficit in the caudal hypothalamus, thus reducing renal sympathetic overactivity and hypertension [8]. Ionotropic glutamate receptors are ligand-gated ion channels mediating excitatory synaptic transmission throughout the central nervous system [22]. These receptors can be classified into three distinct families. Of these receptors, GRIN 2B encodes one of the subunits of the NMDA receptor [22]. We here found that prenatal LPS exposure increased GRIN2B protein expression, whereas decreased GAD65 protein expression in the PVN of the offspring rats. Prenatal LPS administration thus caused an imbalance between excitatory and inhibitory neurons, which caused overexcitation of sympathetic outflow and hypertension in the offspring rats.

ROS has a crucial role in the pathophysiology of hypertension and various kidney disorders [1]. Oxidative stress mediates much of the effect of the activated RAS to augment kidney damage and to activate the brain sympathetic outflow [20]. By binding with Ang II, AT1 triggers NADPH-oxidase activation, inducing ROS generation and transitioning the microglia from the immunoregulatory M2 phenotype to the proinflammatory M1 phenotype [18]. Oxidative stress in the brain or kidney modulates the sympathetic tone [23]. Prenatal LPS exposure triggered an increase in ROS levels and AT1R expression within the PVN and overactivation of the sympathetic outflow. This evidenced that the interaction among ROS levels, RAS, and SNS promoted hypertension progression.

How the adverse stimulative information during pregnancy is transmitted to the next generation and how the RAS system remains active during development remain unclear. Epigenetics refers to inheritable changes in the cellular phenotype without any alteration in the DNA sequence [24], including DNA methylation, histone modification, chromosome remodeling, and regulation of non-coding RNA [25]. During pregnancy, adverse stimuli may be passed from the parent to the child through epigenetic inheritance. For example, a low-protein diet or dexamethasone intervention during pregnancy can cause increased AT1R gene expression in the central PVN region of offspring, accompanied by decreased methylation of the AT1R gene [26, 27]. Low birth weight activates RAS, resulting in changes of AT1R protein expression, and acetylation of histone H3K9 of the AT1R promoter in the hearts of lambs in early postnatal life [28]. In this study, we found that prenatal LPS stimulation caused H3 acetylation at H3K9 and H3K14 (no significant difference was found in H3K27ac) in PVN, which might indicate that LPS, via regulation of epigenetic markers, such as H3K9ac and H3K14, affects AT_1_R expression, which needs to be determined in the future.

The sodium excretion is regulated by numerous factors, including SNS and RAS system. Renal nerve stimulation enhances (Na, K)-ATPase activity and promotes proximal tubule sodium and water reabsorption. Previous studies in genetic models of hypertension have supported the role for the renal adrenergic system in the sodium retention which precedes the development of hypertension in spontaneous hypertensive rats [29]. Our present study indicated that the sympathetic system activity is increased in the offsprings, therefore, sodium reabsorption would be increased, leading to lower sodium excretion and higher potassium excretion due to the sodium-potassium exchange. After melatonin treatment, the sympathetic activity would be alleviated, therefore, the sodium excretion would be recovered to some degrees.

In conclusion, prenatal LPS exposure leads to increased RAS expression within the PVN and overactivation of sympathetic outflow, thereby contributing to hypertension in offspring rats. Histone acetylation is involved in transmitting adverse information to future generations. Melatonin is expected to be a promising agent for preventing prenatal LPS exposure-induced hypertension.

## References

[1] Sinha N, Dabla PK. Oxidative stress and antioxidants in hypertension-a current review. Curr Hypertens Rev. 2015;11:132–42. 26022210 10.2174/1573402111666150529130922

[2] McMillen IC, Robinson JS. Developmental origins of the metabolic syndrome: prediction, plasticity, and programming. Physiol Rev. 2005;85:571–633. 15788706 10.1152/physrev.00053.2003

[3] de Brito Alves JL, Nogueira VO, Cavalcanti Neto MP, Leopoldino AM, Curti C, et al. Maternal protein restriction increases respiratory and sympathetic activities and sensitizes peripheral chemoreflex in male rat offspring. J Nutr. 2015;145:907–14. 25934662 10.3945/jn.114.202804 PMC6619683

[4] do Nascimento LCP, Neto JPRC, de Andrade Braga V, Lagranha CJ, de Brito Alves JL. Maternal exposure to high-fat and high-cholesterol diet induces arterial hypertension and oxidative stress along the gut-kidney axis in rat offspring. Life Sci. 2020;261:118367. 32882266 10.1016/j.lfs.2020.118367

[5] Celsi G, Kistner A, Aizman R, Eklöf AC, Ceccatelli S, et al. Prenatal dexamethasone causes oligonephronia, sodium retention, and higher blood pressure in the offspring. Pediatr Res. 1998;44:317–22. 9727707 10.1203/00006450-199809000-00009

[6] Wang J, Cui J, Chen R, Deng Y, Liao X, et al. Prenatal Exposure to Lipopolysaccharide Alters Renal DNA Methyltransferase Expression in Rat Offspring. PLoS One. 2017;12:e0169206. 28103274 10.1371/journal.pone.0169206 PMC5245821

[7] Nishi EE, Almeida VR, Amaral FG, Simon KA, Futuro-Neto HA, et al. Melatonin attenuates renal sympathetic overactivity and reactive oxygen species in the brain in neurogenic hypertension. Hypertens Res. 2019;42:1683–91. 31316170 10.1038/s41440-019-0301-z

[8] Ferreira-Junior NC, Ruggeri A, Silva SD Jr, Zampieri TT, Ceroni A, Michelini LC. Exercise training increases GAD65 expression, restores the depressed GABAA receptor function within the PVN and reduces sympathetic modulation in hypertension. Physiol Rep. 2019;7:e14107. 31264387 10.14814/phy2.14107 PMC6603325

[9] de Almeida Chaves Rodrigues AF, de Lima IL, Bergamaschi CT, Campos RR, Hirata AE, et al. Increased renal sympathetic nerve activity leads to hypertension and renal dysfunction in offspring from diabetic mothers. Am J Physiol Ren Physiol. 2013;304:F189–97. 10.1152/ajprenal.00241.2012 23136005

[10] Samuelsson AM, Morris A, Igosheva N, Kirk SL, Pombo JM, et al. Evidence for sympathetic origins of hypertension in juvenile offspring of obese rats. Hypertension. 2010;55:76–82. 19901159 10.1161/HYPERTENSIONAHA.109.139402

[11] Shanks J, Ramchandra R. Angiotensin II and the Cardiac Parasympathetic Nervous System in Hypertension. Int J Mol Sci. 2021;22:12305. 34830184 10.3390/ijms222212305 PMC8624735

[12] Chao YM, Wu KLH, Tsai PC, Tain YL, Leu S, et al. Anomalous AMPK-regulated angiotensin AT_1_R expression and SIRT1-mediated mitochondrial biogenesis at RVLM in hypertension programming of offspring to maternal high fructose exposure. J Biomed Sci. 2020;27:68. 32446297 10.1186/s12929-020-00660-z PMC7245869

[13] Chen K, Sun D, Qu S, Chen Y, Wang J, et al. Prenatal cold exposure causes hypertension in offspring by hyperactivity of the sympathetic nervous system. Clin Sci. 2019;133:1097–113. 10.1042/CS20190254 PMC6833955 31015358

[14] Azedi F, Mehrpour M, Talebi S, Zendedel A, Kazemnejad S, et al. Melatonin regulates neuroinflammation ischemic stroke damage through interactions with microglia in reperfusion phase. Brain Res. 2019;1723:146401. 31445031 10.1016/j.brainres.2019.146401

[15] Tan DX, Manchester LC, Esteban-Zubero E, Zhou Z, Reiter RJ. Melatonin as a Potent and Inducible Endogenous Antioxidant: Synthesis and Metabolism. Molecules. 2015;20:18886–906. 26501252 10.3390/molecules201018886 PMC6332205

[16] Campos RR, Oliveira-Sales EB, Nishi EE, Paton JF, Bergamaschi CT. Mechanisms of renal sympathetic activation in renovascular hypertension. Exp Physiol. 2015;100:496–501. 25639235 10.1113/expphysiol.2014.079855

[17] de Kloet AD, Krause EG, Shi PD, Zubcevic J, Raizada MK, et al. Neuroimmune communication in hypertension and obesity: a new therapeutic angle? Pharm Ther. 2013;138:428–40. 10.1016/j.pharmthera.2013.02.005 PMC3646992 23458610

[18] Cui C, Xu P, Li G, Qiao Y, Han W, et al. Vitamin D receptor activation regulates microglia polarization and oxidative stress in spontaneously hypertensive rats and angiotensin II-exposed microglial cells: Role of renin-angiotensin system. Redox Biol. 2019;26:101295. 31421410 10.1016/j.redox.2019.101295 PMC6831892

[19] Hao XQ, Zhang HG, Yuan ZB, Yang DL, Hao LY, Li XH. Prenatal exposure to lipopolysaccharide alters the intrarenal renin-angiotensin system and renal damage in offspring rats. Hypertens Res. 2010;33:76–82. 19911002 10.1038/hr.2009.185

[20] Cao W, Li A, Li J, Wu C, Cui S, et al. Reno-Cerebral Reflex Activates the Renin-Angiotensin System, Promoting Oxidative Stress and Renal Damage After Ischemia-Reperfusion Injury. Antioxid Redox Signal. 2017;27:415–32. 28030955 10.1089/ars.2016.6827 PMC5549812

[21] Jiang W, Kakizaki T, Fujihara K, Miyata S, Zhang Y, et al. Impact of GAD65 and/or GAD67 deficiency on perinatal development in rats. FASEB J. 2022;36:e22123. 34972242 10.1096/fj.202101389R

[22] Myers SJ, Yuan H, Kang JQ, Tan FCK, Traynelis SF, Low CM. Distinct roles of *GRIN2A* and *GRIN2B* variants in neurological conditions. F1000Res. 2019;8:F1000 Faculty Rev-1940. 31807283 10.12688/f1000research.18949.1 PMC6871362

[23] Kreuz S, Fischle W. Oxidative stress signaling to chromatin in health and disease. Epigenomics. 2016;8:843–62. 27319358 10.2217/epi-2016-0002 PMC5619053

[24] Zelko IN, Zhu J, Roman J. Maternal undernutrition during pregnancy alters the epigenetic landscape and the expression of endothelial function genes in male progeny. Nutr Res. 2019;61:53–63. 30683439 10.1016/j.nutres.2018.10.005 PMC6355254

[25] Torres-Zelada EF, George S, Blum HR, Weake VM. Chiffon triggers global histone H3 acetylation and expression of developmental genes in Drosophila embryos. J Cell Sci. 2022;135:jcs259132. 34908116 10.1242/jcs.259132 PMC8917357

[26] Kawakami-Mori F, Nishimoto M, Reheman L, Kawarazaki W, Ayuzawa N, et al. Aberrant DNA methylation of hypothalamic angiotensin receptor in prenatal programmed hypertension. JCI Insight. 2018;3:e95625. 30385711 10.1172/jci.insight.95625 PMC6238751

[27] Liu Y, Liang M. Functional role of epigenetic regulation in the development of prenatal programmed hypertension. Kidney Int. 2019;96:10–12. 31229025 10.1016/j.kint.2019.03.003

[28] Wang KC, Brooks DA, Summers-Pearce B, Bobrovskaya L, Tosh DN, et al. Low birth weight activates the renin-angiotensin system, but limits cardiac angiogenesis in early postnatal life. Physiol Rep. 2015;3:e12270. 25649246 10.14814/phy2.12270 PMC4393187

[29] Beach RE, DuBose TD Jr. Adrenergic regulation of (Na+, K+)-ATPase activity in proximal tubules of spontaneously hypertensive rats. Kidney Int. 1990;38:402–8. 2172614 10.1038/ki.1990.219

